# Supplementary material for: From emergence to endemicity of highly pathogenic H5 avian influenza viruses in Taiwan
Source: Nat Commun. 2024 Oct 29;15:9348. doi: 10.1038/s41467-024-53816-y (PMC11522503; doi:10.1038/s41467-024-53816-y)
Supplement: Supplementary file 1 — Supplementary Information [file 41467_2024_53816_MOESM1_ESM.pdf]

From emergence to endemicity of highly pathogenic H5 avian influenza viruses in Taiwan

Supplementary Information

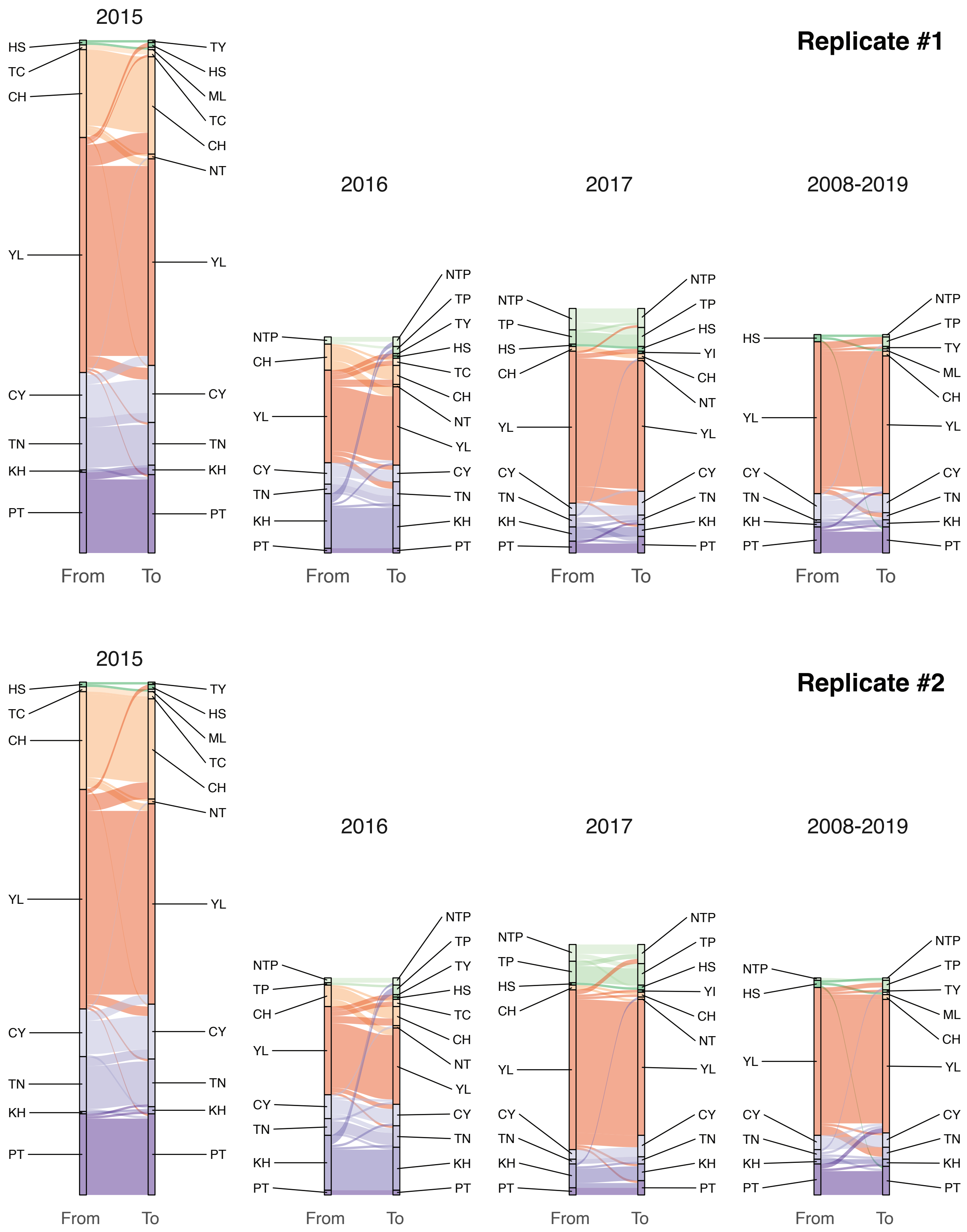

Supplementary Figure 1. Visualisation of the dispersal pattern of the clade 2.3.4.4c virus in discrete geographical divisions. The connected lines represent branches inferred by the continuous phylogeographic approach (see Figure 3), with origin and destination locations determined as county or city-level divisions. The lines were coloured based on the origin. Replicated results from parallel runs are shown.

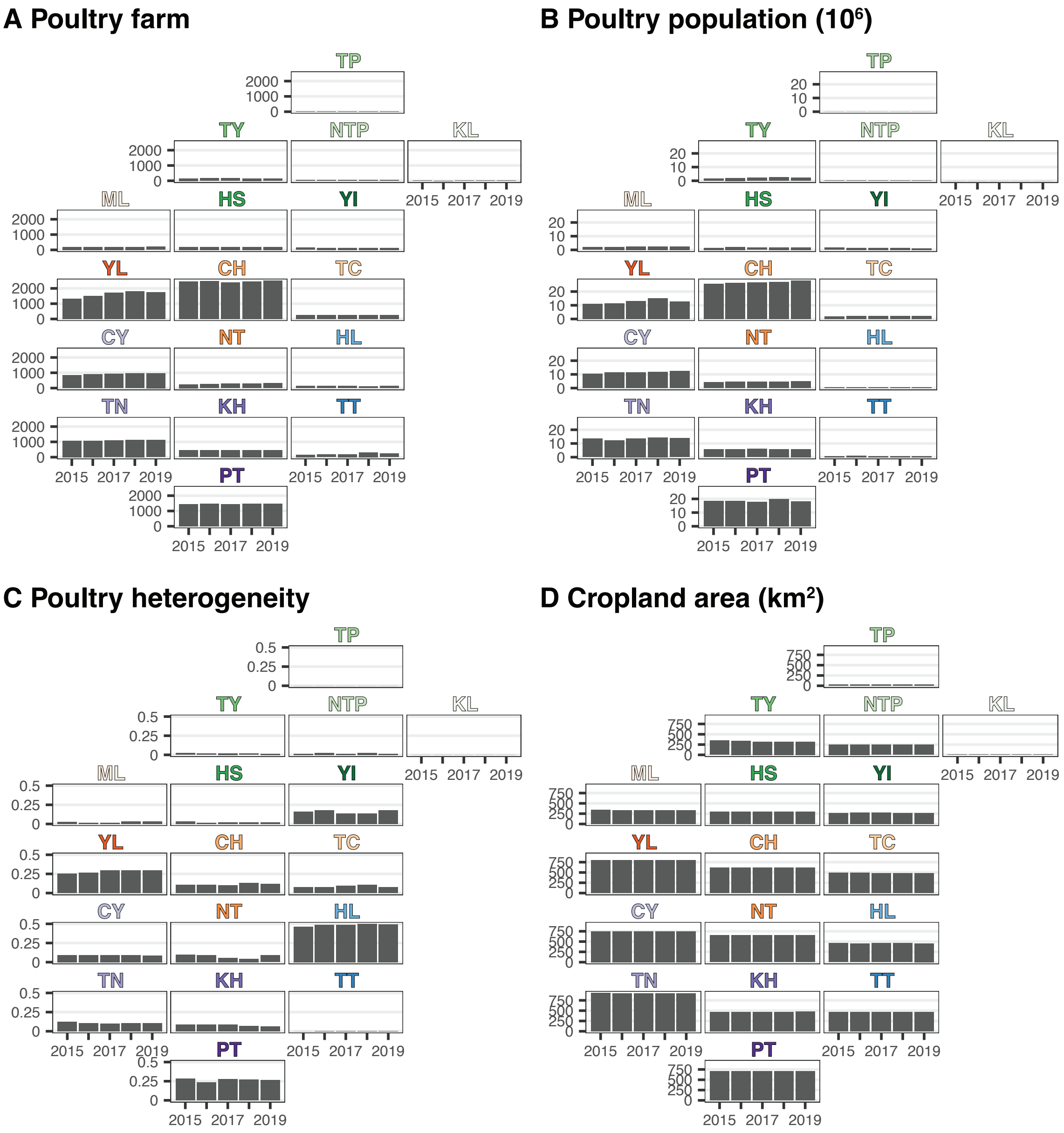

Supplementary Figure 2. Agricultural characteristics of county or city-level administrative areas in Taiwan.

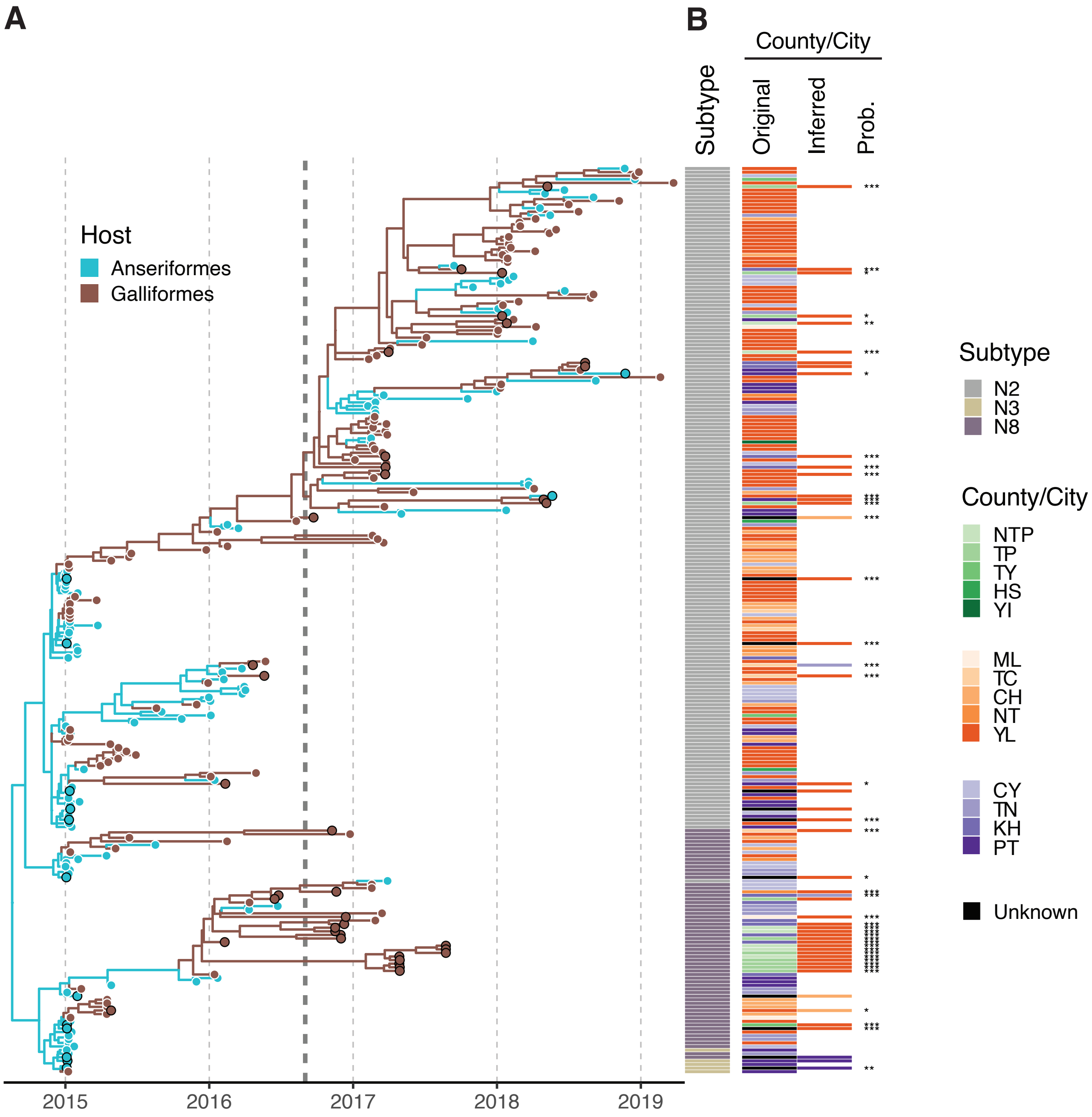

Supplementary Figure 3. Summary of the host and geographical information associated with the genomic data used in this study. (A) The time-scaled phylogeny was reconstructed using the HA genes of the clade 2.3.4.4c virus in Taiwan. The tips on the phylogeny are colored according to the host genera, Galliformes (chicken, turkey and quail) or Anseriformes (duck and goose), logged in the sequence metadata. The branches are colored according to the states inferred on the summarized maximum clade credibility (MCC) tree. The tips with black borders indicate taxa that have uncertain state assignments in geographical analyses. The vertical dashed line denotes September 1st, 2016. (B) The adjacent heatmap shows the NA subtypes and the corresponding collection locations of the tree taxa. The discrete diffusion model was used to estimate the uncertain geographical locations and posterior probabilities. Only the results of samples collected in slaughterhouses/rendering factories and samples lacking geospatial information, which were implemented with state uncertainty, are shown in the 'Inferred' column. The symbols *, ** and *** denote posterior probability>0.7, 0.8 and 0.9, respectively.

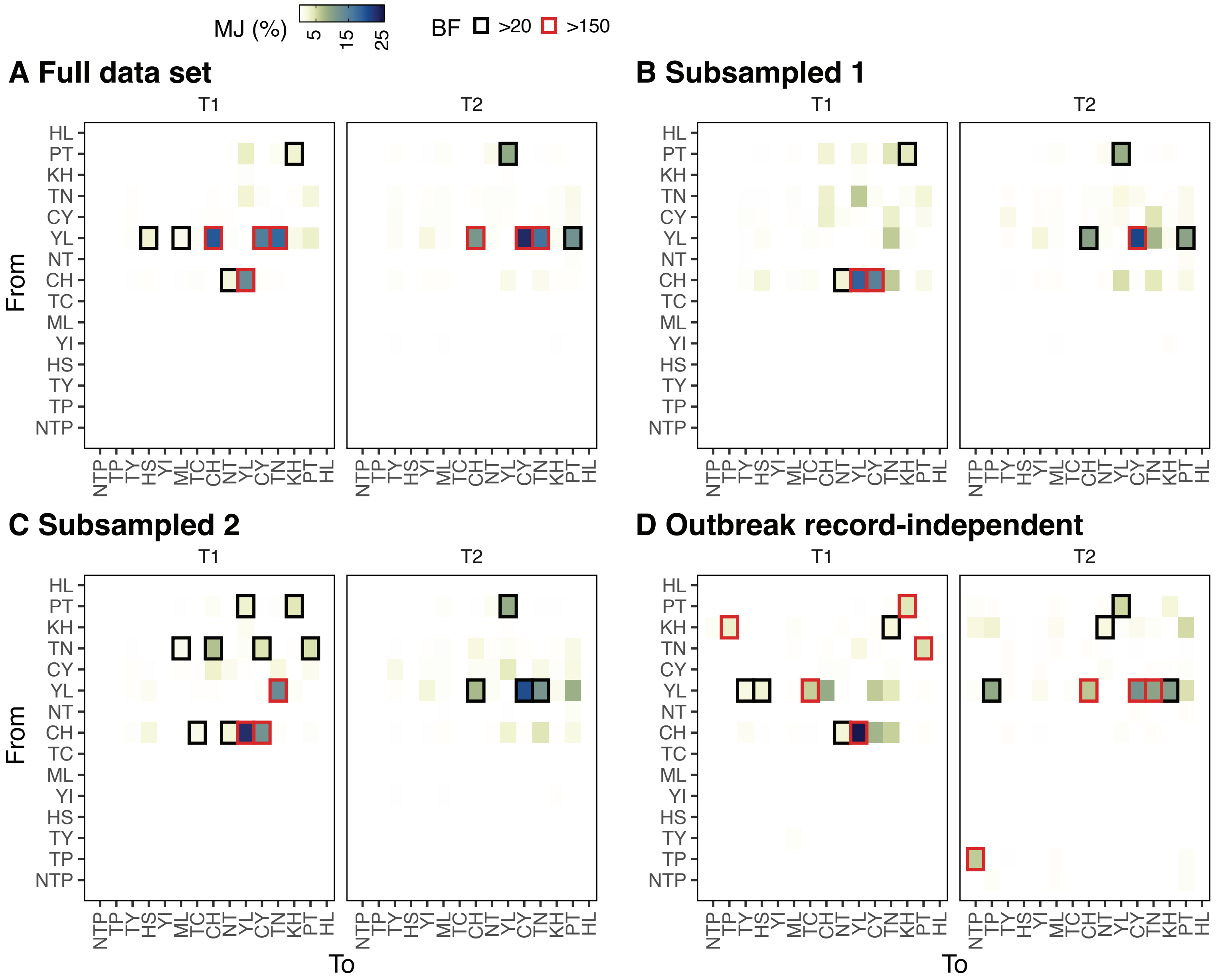

Supplementary Figure 4. Estimated number of transition events between counties/cities in Taiwan inferred by the discrete phylogeographic method. The color of the heatmap reflects the proportions of between-locations Markov jumps to the total jumps in the distinct time period. The directions supported by Bayesian factor (BF) are highlighted by black (>20) or red (>150) edges. Results of a full genomic data set (A), along with two data sets with reduced samples from county YL are shown (B and C). Using the same genomic data as panel (A), Bayesian inference was performed for panel (D) with geographical states assigned independently of outbreak records. The locations were determined by the sequence metadata if available, or using a uniform prior over all isolated counties/cities if the collection site was unknown.

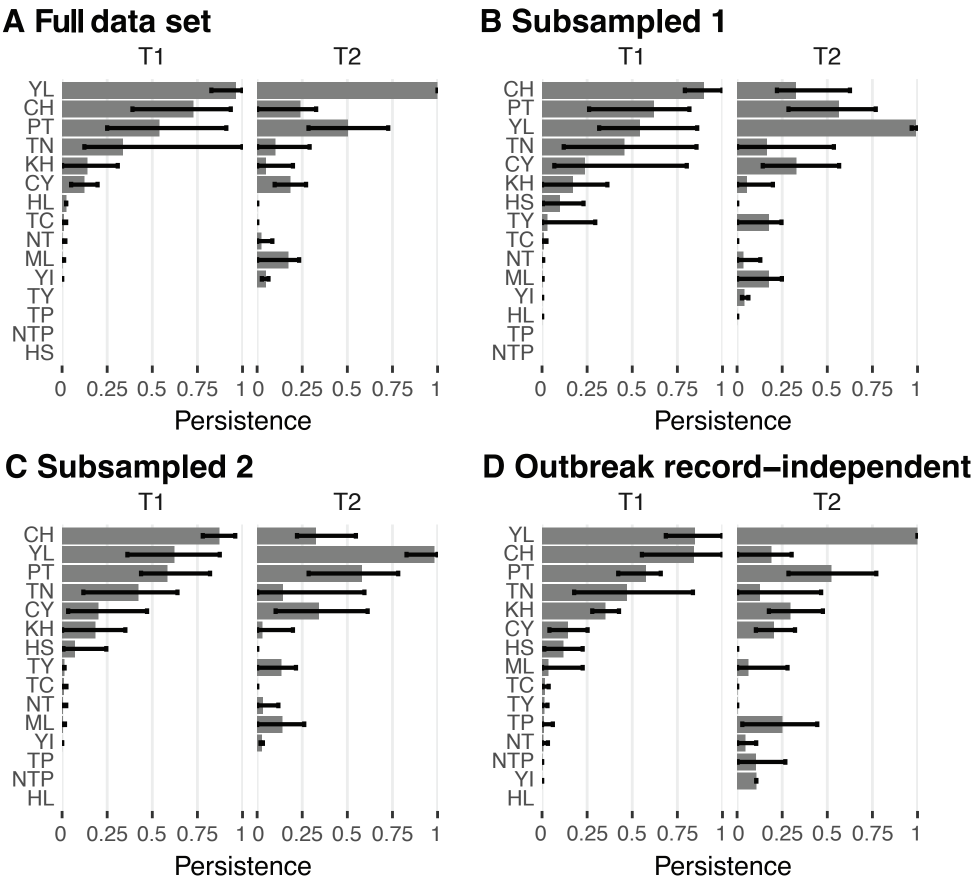

Supplementary Figure 5. Persistence of the virus assessed by the discrete phylogeographic method. To calculate the value for each location, the branch length was unified from branches where both nodes were estimated to share the same state. The unified time interval was presented as a proportion divided by the time span of T1/T2. The mean and 95% HPD of the proportions summarized by 1000 posterior trees are indicated by the bars. The results of the four panels were obtained from the same MCMC runs as the corresponding panels in Supplementary Figure 4.

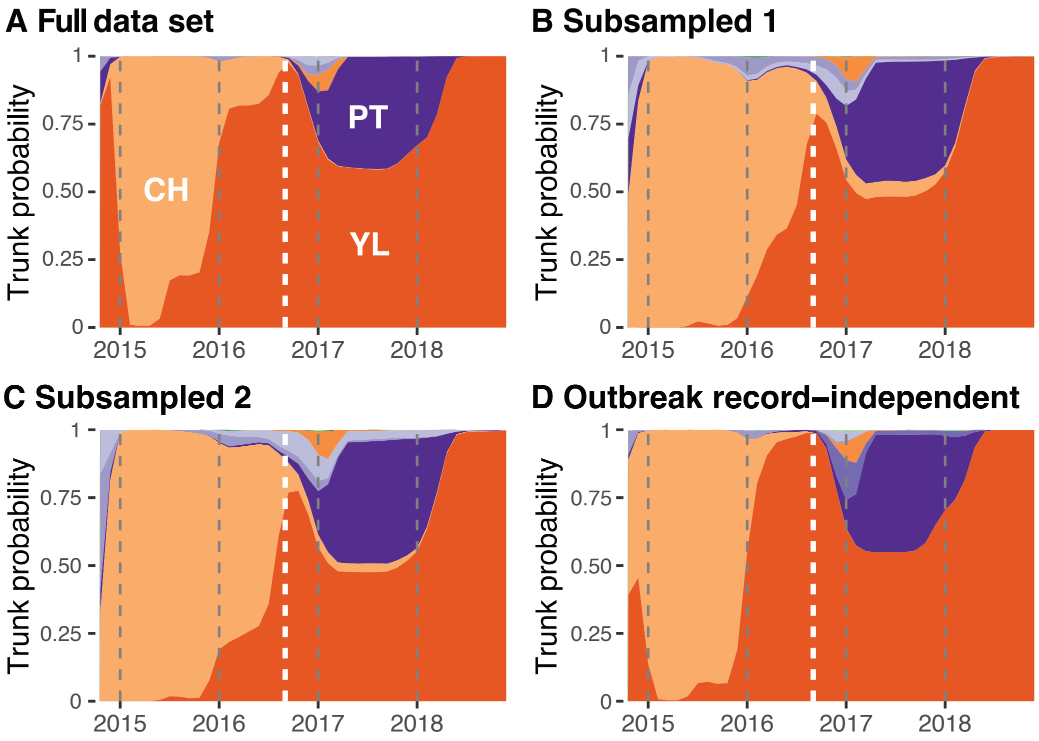

Supplementary Figure 6. Inferred trunk locations of Taiwan clade 2.3.4.4c phylogenies through time in different genetic data sets (A-C) or with a simple geographic state assignment scheme (D). The proportions at each time point indicate the posterior support for viruses circulating in a particular county/city occupying the trunk of the tree. The areas are colored using the same scheme as Figure 2. The white dashed lines indicate the boundary of T1 and T2, September 1st, 2016.

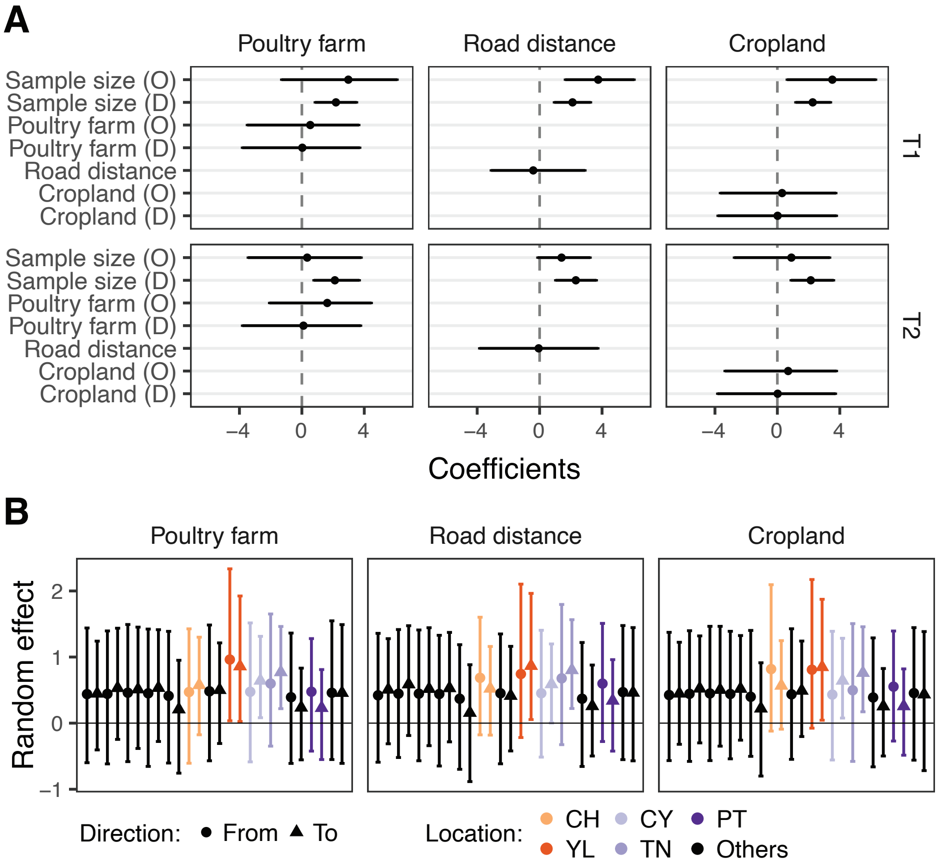

Supplementary Figure 7. Evaluating predictors in reduced models of time-heterogeneous phylogenetic GLM. (A) The conditional effect sizes in models containing respective predictors. The predictor names are denoted by O in parentheses for origin and D for destination. (B) Location-specific random effects in the three models, with high incidence areas colored. The estimates are in log space and presented as mean with 95% HPD interval.

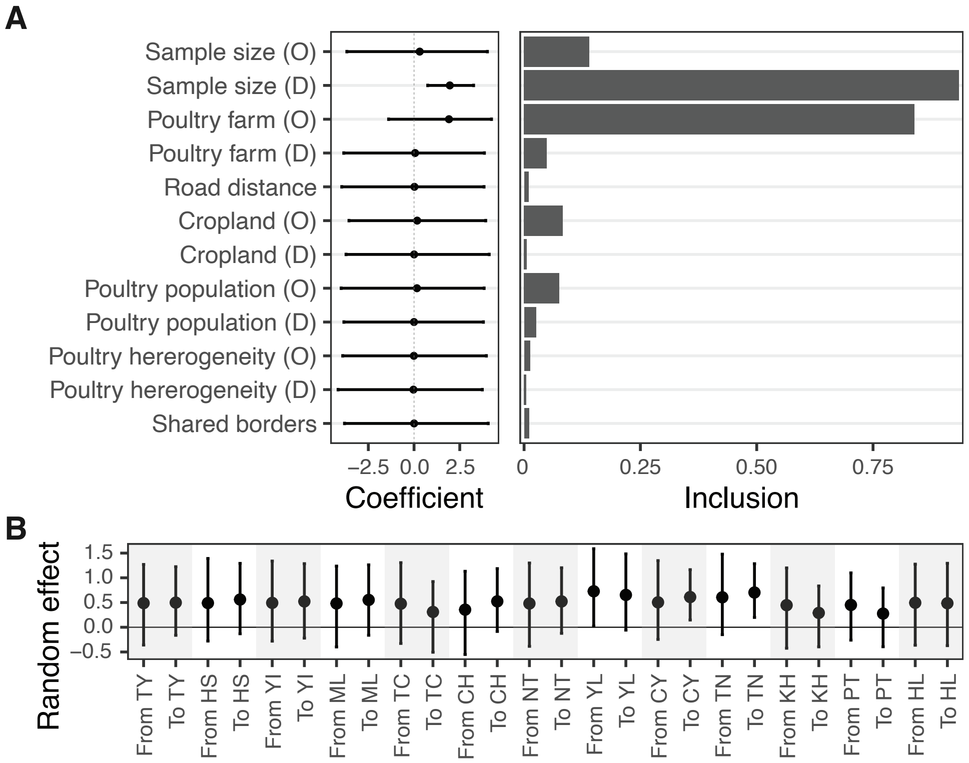

Supplementary Figure 8. Evaluating predictors in a time-homogeneous model. (A) The conditional effect sizes and the inclusion probabilities of predictors estimated by the conventional phylogenetic GLM models. The predictor names are denoted by O in parentheses for origin and D for destination. (B) Location-specific random effects in the GLM model. The estimates are in log space and presented as mean with 95% HPD interval.

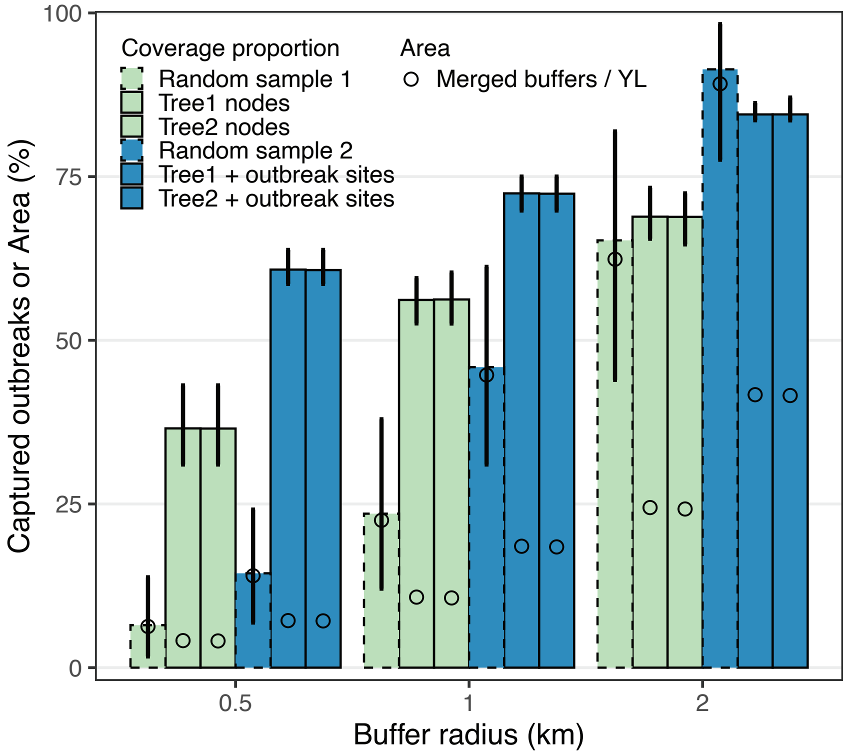

Supplementary Figure 9. Impact of buffer radius on evaluating the re-emergence of new outbreaks in county YL. The estimated locations of tree nodes using the continuous phylogenetic method during T2, combined with or without contemporary outbreak sites, served as central points to create buffers with different radius distances on the map. The proportion of new outbreak sites covered by the buffer areas was calculated for each posterior tree. These outbreak sites were reported between 2019 and 2022, after the latest available genetic data. Null models were created by randomly distributing sites in YL with the same number of tree nodes (Random sample 1, green) or tree nodes plus outbreak sites (Random sample 2, blue). The bars show the mean values of two parallel continuous phylogeographic analyses each with 1000 posterior trees, and null models. Error bars indicate 95% credible intervals. The open circle in each bar represents the ratio of the area of merged buffers to the area of YL.

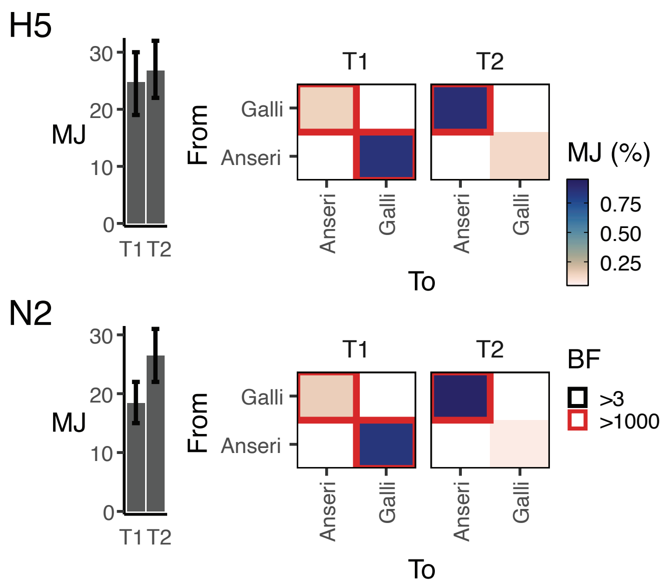

Supplementary Figure 10. Diffusion between host groups. Markov jumps (MJ) and Bayes factors (BF) were estimated by the discrete phylogenetic method. Total MJ counts in different time spans are shown on the left. The heatmaps present the jumps as proportions to the total counts. Anseri refers to the Anseriformes genus and Galli refers to the Galliformes genus. Results based on both HA (H5) and NA (N2) are shown.

Supplementary Table 1. Selection pressure for the surface proteins of H5 avian influenza lineages in Taiwan.

	H5		N2	
	GsGd Clade 2.3.4.4	North American	GsGd Clade 2.3.4.4	North American
dN/dS (ω) (95% CI)	0.204 (0.180-0.230)	0.207 (0.174-0.245)	0.355 (0.316-0.397)	0.233 (0.192-0.279)
Pervasive positively selected sites	131	-	77	-
Episodic positively selected sites	131, 387, 403, 545	17, 102, 137, 155, 317	77, 313, 416, 439	5, 6, 261, 307
No. of fixed sites among selected sites	0	0	0	0

Supplementary Table 2. List of database accession numbers from GenBank and GISAID.

	accession no.	strain name
1	MW333030	duck/Nantou/15A03445-11-20T/2015
2	MW333038	chicken/Changhua/15010120-1/2015
3	MW333046	chicken/Changhua/15010288/2015
4	MW333054	chicken/Changhua/15010324-2/2015
5	MW333062	chicken/Changhua/15010361-1/2015
6	MW333070	chicken/Changhua/15010362-2/2015
7	MW333078	chicken/Changhua/15010363-2/2015
8	MW333086	chicken/Changhua/15040036-1/2015
9	MW333094	chicken/Changhua/15040043-1/2015
10	MW333102	chicken/Changhua/15040037-2/2015
11	MW333110	chicken/Changhua/15040042/2015
12	MW333118	chicken/Changhua/15050013-1/2015
13	MW333126	chicken/Changhua/15050038-1/2015
14	MW333134	chicken/Changhua/15060027-2/2015
15	MW333142	chicken/Changhua/15060029/2015
16	MW333150	chicken/Changhua/15060037-1/2015
17	MW333158	chicken/Changhua/15120008/2015
18	MW333166	chicken/Changhua/16010060-2/2016
19	MW333174	chicken/Changhua/16020042-2/2016
20	MW333182	chicken/Changhua/17030063-1/2017
21	MW333190	chicken/Changhua/17060006/2017
22	MW333198	chicken/Changhua/18010011-1/2018
23	MW333206	chicken/Changhua/18040008-1/2018
24	MW333214	chicken/Chiayi/15010287-2/2015
25	MW333222	chicken/Chiayi/15010409-2/2015
26	MW333230	chicken/Chiayi/15010454-1/2015
27	MW333238	chicken/Chiayi/17030059-2/2017
28	MW333246	chicken/Chiayi/17040009-2/2017
29	MW333254	chicken/Chiayi/18120003-1/2018
30	MW333262	chicken/Hsinchu/16080004-2/2016
31	MW333270	chicken/Kaohsiung/16010047/2016
32	MW333278	chicken/Kaohsiung/16020017-2/2016
33	MW333294	chicken/Kaohsiung/16070003/2016
34	MW333302	chicken/Kaohsiung/16120012/2016
35	MW333310	chicken/Kaohsiung/16120034/2016
36	MW333326	chicken/Kaohsiung/17030023/2017
37	MW333334	chicken/Kaohsiung/17040006-2/2017
38	MW333342	chicken/Kaohsiung/17040007/2017
39	MW333350	chicken/Kaohsiung/17100002/2017
40	MW333358	chicken/Kaohsiung/18080004-1/2018
41	MW333366	chicken/Kaohsiung/18080004-2/2018
42	MW333374	chicken/Miaoli/15010421-1/2015
43	MW333382	chicken/Miaoli/18040011-1/2018
44	MW333390	chicken/Nantou/16110023-1/2016
45	MW333398	chicken/New_Taipei_City/16110019-1/2016
46	MW333406	chicken/New_Taipei_City/16110020-2/2016
47	MW333414	chicken/New_Taipei_City/17040002-2/2017
48	MW333422	chicken/New_Taipei_City/17050004-2/2017
49	MW333430	chicken/New_Taipei_City/17090001/2017
50	MW333454	chicken/New_Taipei_City/18010021-3/2018
51	MW333462	chicken/Pingtung/15010402/2015
52	MW333470	chicken/Pingtung/15050006-1/2015
53	MW333478	chicken/Pingtung/16020009-3/2016
54	MW333486	chicken/Pingtung/18010017-1/2018
55	MW333494	chicken/Pingtung/18010019-2/2018
56	MW333502	chicken/Pingtung/18020015-1/2018
57	MW333510	chicken/Pingtung/18050006-1/2018
58	MW333518	chicken/Pingtung/18080001-1/2018
59	MW333526	chicken/Taichung/15010399/2015
60	MW333534	chicken/Taichung/16040032-3/2016
61	MW333542	chicken/Taichung/16050044-2/2016
62	MW333550	chicken/Taichung/16110017-3/2016
63	MW333558	chicken/Tainan/15020214/2015
64	MW333566	chicken/Tainan/16040035-1/2016
65	MW333574	chicken/Tainan/17030058-1/2017

66	MW333582	chicken/Tainan/18040014-2/2018
67	MW333590	chicken/Taipei_City/17090004/2017
68	MW333598	chicken/Taipei_City/18010007-3/2018
69	MW333606	chicken/Taipei_City/18010008-3/2018
70	MW333614	chicken/Taipei_City/18050007-1/2018
71	MW333622	chicken/Taipei_City/18050010-8/2018
72	MW333646	chicken/Taipei/16120021/2016
73	MW333654	chicken/Taipei/17050005-1/2017
74	MW333662	chicken/Taipei/17050006-2/2017
75	MW333686	chicken/Taipei/17050009-3/2017
76	MW333694	chicken/Yunlin/15020048-1/2015
77	MW333702	chicken/Yunlin/15040002-2/2015
78	MW333710	chicken/Yunlin/15040027/2015
79	MW333718	chicken/Yunlin/15050012/2015
80	MW333726	chicken/Yunlin/15050037-2/2015
81	MW333734	chicken/Yunlin/15050039-2/2015
82	MW333742	chicken/Yunlin/15060014-1/2015
83	MW333750	chicken/Yunlin/15080024/2015
84	MW333758	chicken/Yunlin/16010001-1/2016
85	MW333766	chicken/Yunlin/16010008-1/2016
86	MW333774	chicken/Yunlin/16020038/2016
87	MW333782	chicken/Yunlin/16050053/2016
88	MW333790	chicken/Yunlin/17010001/2017
89	MW333798	chicken/Yunlin/17010014/2017
90	MW333806	chicken/Yunlin/17020033-2/2017
91	MW333814	chicken/Yunlin/17020094-1/2017
92	MW333822	chicken/Yunlin/17020095-2/2017
93	MW333830	chicken/Yunlin/17030001-1/2017
94	MW333838	chicken/Yunlin/17030007-1/2017
95	MW333846	chicken/Yunlin/17030010-1/2017
96	MW333854	chicken/Yunlin/17030014-1/2017
97	MW333862	chicken/Yunlin/17030020-2/2017
98	MW333870	chicken/Yunlin/17030021-1/2017
99	MW333878	chicken/Yunlin/17030032-2/2017
100	MW333886	chicken/Yunlin/17030065-2/2017
101	MW333894	chicken/Yunlin/17040003-1/2017
102	MW333902	chicken/Yunlin/17040004-2/2017
103	MW333910	chicken/Yunlin/17040005-1/2017
104	MW333918	chicken/Yunlin/17040010-1/2017
105	MW333926	chicken/Yunlin/17040011-1/2017
106	MW333934	chicken/Yunlin/17040012-1/2017
107	MW333942	chicken/Yunlin/17060019/2017
108	MW333950	chicken/Yunlin/17070010/2017
109	MW333958	chicken/Yunlin/18010001-2/2018
110	MW333966	chicken/Yunlin/18010003-3/2018
111	MW333974	chicken/Yunlin/18010023-1/2018
112	MW333982	chicken/Yunlin/18010024-2/2018
113	MW333990	chicken/Yunlin/18020003-2/2018
114	MW333998	chicken/Yunlin/18020004-1/2018
115	MW334006	chicken/Yunlin/18020005-2/2018
116	MW334014	chicken/Yunlin/18020008-2/2018
117	MW334022	chicken/Yunlin/18020021-1/2018
118	MW334030	chicken/Yunlin/18030002-1/2018
119	MW334038	chicken/Yunlin/18040006-3/2018
120	MW334046	chicken/Yunlin/18050013-1/2018
121	MW334054	chicken/Yunlin/18060001-2/2018
122	MW334062	chicken/Yunlin/18070003-3/2018
123	MW334070	chicken/Yunlin/18080006-1/2018
124	MW334078	duck/Changhua/15A03431-1-20T/2015
125	MW334086	duck/Changhua/15A03473-1-20T/2015
126	MW334094	duck/Chiayi/15080023/2015
127	MW334102	duck/Hsinchu/15020216/2015
128	MW334110	duck/Kaohsiung/15010213/2015
129	MW334118	duck/Nantou/15A3437/2015
130	MW334126	duck/Nantou/17A0243/2017
131	MW334134	duck/Pingtung/15010145/2015
132	MW334142	duck/Pingtung/15010157-1/2015
133	MW334150	duck/Pingtung/15010180/2015
134	MW334158	duck/Pingtung/15010444/2015
135	MW334166	duck/Pingtung/15010461-1/2015

136	MW334174	duck/Pingtung/15120006/2015
137	MW334182	duck/Pingtung/15A03399-1-10T/2015
138	MW334190	duck/Pingtung/17A00408-1-10T/2017
139	MW334198	duck/Pingtung/17A0160/2017
140	MW334206	duck/Pingtung/18A00003-11-20T/2018
141	MW334214	duck/Pingtung/18A00076-11-20T/2018
142	MW334222	duck/Pingtung/18X00077/2018
143	MW334230	duck/Taichung/15010400-1/2015
144	MW334238	duck/Tainan/15010403/2015
145	MW334262	duck/Taoyuan/16010023-1/2016
146	MW334270	duck/Yilan/17A0092/2017
147	MW334278	duck/Yunlin/15010115-2/2015
148	MW334286	duck/Yunlin/15010244-1/2015
149	MW334294	duck/Yunlin/15010277/2015
150	MW334302	duck/Yunlin/15010310-4/2015
151	MW334310	duck/Yunlin/15010318-2/2015
152	MW334318	duck/Yunlin/15010369-1/2015
153	MW334326	duck/Yunlin/15010458-1/2015
154	MW334334	duck/Yunlin/15010538/2015
155	MW334342	duck/Yunlin/15070002-2/2015
156	MW334350	duck/Yunlin/15A03403-1-20T/2015
157	MW334358	duck/Yunlin/15A04883-1-10T/2015
158	MW334366	duck/Yunlin/15A5019/2015
159	MW334374	duck/Yunlin/17110001/2017
160	MW334382	duck/Yunlin/17A0086/2017
161	MW334390	duck/Yunlin/17A0686/2017
162	MW334398	duck/Yunlin/18010004-2/2018
163	MW334406	duck/Yunlin/18060006-1/2018
164	MW334414	duck/Yunlin/18060007-3/2018
165	MW334422	duck/Yunlin/18X00007-3/2018
166	MW334446	goose/Changhua/15040008/2015
167	MW334454	goose/Chiayi/15010008-3/2015
168	MW334462	goose/Chiayi/15010016-1/2015
169	MW334470	goose/Chiayi/15020022/2015
170	MW334478	goose/Chiayi/16010017-2/2016
171	MW334486	goose/Chiayi/16040003-1/2016
172	MW334494	goose/Chiayi/16040005/2016
173	MW334502	goose/Chiayi/16040007-1/2016
174	MW334510	goose/Chiayi/17020027-1/2017
175	MW334518	goose/Chiayi/17040014/2017
176	MW334526	goose/Chiayi/18010006/2018
177	MW334534	goose/Chiayi/18020002-2/2018
178	MW334566	goose/Pingtung/15010007-4/2015
179	MW334574	goose/Pingtung/15010037-1/2015
180	MW334582	goose/Pingtung/15050014/2015
181	MW334590	goose/Pingtung/16010061/2016
182	MW334598	goose/Tainan/16010031-2/2016
183	MW334606	goose/Tainan/16020041-2/2016
184	MW334614	goose/Tainan/16040018-1/2016
185	MW334622	goose/Tainan/16070002/2016
186	MW334630	goose/Tainan/17030022-1/2017
187	MW334638	goose/Tainan/17030024-1/2017
188	MW334646	goose/Tainan/18010022-1/2018
189	MW334654	goose/Tainan/18050014-1/2018
190	MW334662	goose/Taoyuan/15010041/2015
191	MW334670	goose/Taoyuan/18120002-2/2018
192	MW334678	goose/Yunlin/15010005/2015
193	MW334686	goose/Yunlin/15010009-1/2015
194	MW334694	goose/Yunlin/15010010-2/2015
195	MW334702	goose/Yunlin/15010011-1/2015
196	MW334710	goose/Yunlin/15010029-1/2015
197	MW334718	goose/Yunlin/15040040/2015
198	MW334726	goose/Yunlin/16020008-4/2016
199	MW334734	goose/Yunlin/16020011-1/2016
200	MW334742	goose/Yunlin/16030042-2/2016
201	MW334750	goose/Yunlin/16040002-2/2016
202	MW334758	goose/Yunlin/17100005/2017
203	MW334782	goose/Yunlin/18030020-3/2018
204	MW334790	goose/Yunlin/18040002-3/2018
205	MW334798	goose/Yunlin/18040018-2/2018

206	MW334806	goose/Yunlin/18050003-2/2018
207	MW334814	goose/Yunlin/18090002-3/2018
208	MW334822	goose/Yunlin/18090006-1/2018
209	MW334830	goose/Yunlin/18110002-3/2018
210	MW334854	turkey/Changhua/16010011-1/2016
211	MW334862	turkey/Chiayi/17020086-2/2017
212	MW334870	turkey/Chiayi/17020092-2/2017
213	MW334878	turkey/Chiayi/18010013-3/2018
214	MW334886	turkey/Yunlin/15030069-1/2015
215	MW334894	turkey/Yunlin/15060049-1/2015
216	MW334902	turkey/Yunlin/18020020-2/2018
217	MW334910	turkey/Yunlin/18070001-2/2018
218	MW334918	turkey/Yunlin/18090003-3/2018
219	MW334926	turkey/Yunlin/18110001-3/2018
220	MW334934	turkey/Yunlin/18120005-2/2018
221	EPI588952	goose/Taiwan/a015/2015
222	EPI588960	duck/Taiwan/a043/2015
223	EPI588976	duck/Taiwan/a068/2015
224	KT388444	goose/Taiwan/TNC1/2015
225	KT388572	goose/Taiwan/TNO3/2015
226	KU646901	goose/Taiwan/01038/2015
227	KU646917	duck/Taiwan/A3400/2015
228	KU646925	goose/Taiwan/01019/2015
229	KU646933	goose/Taiwan/01026/2015
230	KU646941	goose/Taiwan/01039/2015
231	KU646861	duck/Taiwan/01006/2015
232	KU646869	goose/Taiwan/01022/2015
233	KU646877	goose/Taiwan/01023/2015
234	KU646885	goose/Taiwan/01031/2015
235	KU646893	goose/Taiwan/01040/2015
236	EPI961922	chicken/Taiwan/u7/2016
237	EPI961933	chicken/Taiwan/x37/2016
238	KP714479	goose/Taiwan/01-003/2015
239	KP714480	goose/Taiwan/01-004/2015
240	KP714481	goose/Taiwan/01-042/2015
241	MW334254	duck/Tainan/16010024-1/2016
242	MW333318	chicken/Kaohsiung/16MB0889/2016
243	MW333638	chicken/Taipei/16060023-1/2016
244	MW333670	chicken/Taipei/17050007-2/2017
245	MW333438	chicken/New_Taipei_City/17090002/2017
246	KU646909	chicken/Taiwan/01174/2015
247	MW334558	goose/Chiayi/18020014-3/2018
248	MW334766	goose/Yunlin/18030020-1/2018
249	MN988773	chicken/Taiwan/A3/2019
250	MN988805	chicken/Taiwan/D9/2019
251	KT388452	goose/Taiwan/TNC2/2015
252	KT388476	goose/Taiwan/TNC5/2015
