# Supplementary material for: From emergence to endemicity of highly pathogenic H5 avian influenza viruses in Taiwan
Source: Nat Commun. 2024 Oct 29;15:9348. doi: 10.1038/s41467-024-53816-y (PMC11522503; doi:10.1038/s41467-024-53816-y)
Supplement: Supplementary file 2 — Reporting Summary [file 41467_2024_53816_MOESM2_ESM.pdf]

nature portfolio

Corresponding author(s): Yao-Tsun Li & Kirstyn Brunker

Last updated by author(s): Oct 16, 2024

Reporting Summary

Nature Portfolio wishes to improve the reproducibility of the work that we publish. This form provides structure for consistency and transparency in reporting. For further information on Nature Portfolio policies, see our Editorial Policies and the Editorial Policy Checklist.

Statistics

For all statistical analyses, confirm that the following items are present in the figure legend, table legend, main text, or Methods section.

n/a	Confirmed	
☐	☒	The exact sample size (*n*) for each experimental group/condition, given as a discrete number and unit of measurement
☐	☒	A statement on whether measurements were taken from distinct samples or whether the same sample was measured repeatedly
☒	☐	The statistical test(s) used AND whether they are one- or two-sided<br>*Only common tests should be described solely by name; describe more complex techniques in the Methods section.*
☐	☒	A description of all covariates tested
☒	☐	A description of any assumptions or corrections, such as tests of normality and adjustment for multiple comparisons
☐	☒	A full description of the statistical parameters including central tendency (e.g. means) or other basic estimates (e.g. regression coefficient) AND variation (e.g. standard deviation) or associated estimates of uncertainty (e.g. confidence intervals)
☐	☒	For null hypothesis testing, the test statistic (e.g. *F*, *t*, *r*) with confidence intervals, effect sizes, degrees of freedom and *P* value noted<br>*Give P values as exact values whenever suitable.*
☐	☒	For Bayesian analysis, information on the choice of priors and Markov chain Monte Carlo settings
☒	☐	For hierarchical and complex designs, identification of the appropriate level for tests and full reporting of outcomes
☐	☒	Estimates of effect sizes (e.g. Cohen's *d*, Pearson's *r*), indicating how they were calculated

Our web collection on statistics for biologists contains articles on many of the points above.

Software and code

Policy information about availability of computer code

Data collection	RSelenium.
Data analysis	Custom scripts used for data analysis are available in the project's GitHub repository. Other analyses use the following software/packages. Nextalign v2.3.0, MAFFT v7.490, Treetime v0.11.1, IQ-TREE v2.2.0, TempEst v1.5.3, BEAST v1.10.4 & v1.10.5, PACT v0.9.4, HyPhy v.2.3.14, R package SERAPHIM, sf.

For manuscripts utilizing custom algorithms or software that are central to the research but not yet described in published literature, software must be made available to editors and reviewers. We strongly encourage code deposition in a community repository (e.g. GitHub). See the Nature Portfolio guidelines for submitting code & software for further information.

Data

Policy information about availability of data

All manuscripts must include a data availability statement. This statement should provide the following information, where applicable:

- Accession codes, unique identifiers, or web links for publicly available datasets
- A description of any restrictions on data availability
- For clinical datasets or third party data, please ensure that the statement adheres to our policy

The accession numbers of the Taiwan sequences analysed in our study are listed in Supplementary Table 2. The XML files required for BEAST, subsampled MCMC trees and summarized results can be found at the project repository (https://github.com/yaotli/endemic).

Research involving human participants, their data, or biological material

Policy information about studies with human participants or human data. See also policy information about sex, gender (identity/presentation), and sexual orientation and race, ethnicity and racism.

Reporting on sex and gender	NA
Reporting on race, ethnicity, or other socially relevant groupings	NA
Population characteristics	NA
Recruitment	NA
Ethics oversight	The study did not involve any human participants.

Note that full information on the approval of the study protocol must also be provided in the manuscript.

Field-specific reporting

Please select the one below that is the best fit for your research. If you are not sure, read the appropriate sections before making your selection.

☐ Life sciences ☐ Behavioural & social sciences ☒ Ecological, evolutionary & environmental sciences

For a reference copy of the document with all sections, see nature.com/documents/nr-reporting-summary-flat.pdf

Ecological, evolutionary & environmental sciences study design

All studies must disclose on these points even when the disclosure is negative.

Study description	Our study comprehensively characterised the dynamics of clade 2.3.4.4c avian influenza virus in Taiwan using phylogenetic methods, with a particular focus on viral dispersal and mechanisms driving endemic transmission after the initial epidemic wave. We used time-heterogeneous models integrated with outbreak information to identify the spatial and ecological sources at different epidemiological stages.
Research sample	The phylogenetic analyses used the genomic sequences of GsGd clade 2.3.4.4c H5 avian influenza virus in Taiwan.
Sampling strategy	The main dataset included all available HA sequences, with the exception of sequences isolated in apparently the same outbreak. Sensitive analyses were performed using subsampled datasets generated by downsampling sequences collected in a particular county.
Data collection	We downloaded all available Taiwanese and international GsGd sequences from NCBI or GISAID. The sequences isolated in Taiwan have all been described in previous studies (including ours). Surveillance data on H5 outbreaks in Taiwan were collected from the BAPHIQ website (https://twai.baphiq.gov.tw/AI/).
Timing and spatial scale	The samples analysed in the study were collected in Taiwan during 2015-2019.
Data exclusions	Genomic sequences of short length or with notable ambiguous nucleotides were removed.
Reproducibility	This study is based on genomic and epidemiological data. The code used to partially reproduce the analytical results and all the data used to generate figures have been shared.
Randomization	NA
Blinding	NA

Did the study involve field work? ☐ Yes ☒ No

Reporting for specific materials, systems and methods

We require information from authors about some types of materials, experimental systems and methods used in many studies. Here, indicate whether each material, system or method listed is relevant to your study. If you are not sure if a list item applies to your research, read the appropriate section before selecting a response.

Materials & experimental systems

n/a	Involved in the study
☒	☐ Antibodies
☒	☐ Eukaryotic cell lines
☒	☐ Palaeontology and archaeology
☒	☐ Animals and other organisms
☒	☐ Clinical data
☒	☐ Dual use research of concern
☒	☐ Plants

Methods

n/a	Involved in the study
☒	☐ ChIP-seq
☒	☐ Flow cytometry
☒	☐ MRI-based neuroimaging

Plants

Seed stocks

Report on the source of all seed stocks or other plant material used. If applicable, state the seed stock centre and catalogue number. If plant specimens were collected from the field, describe the collection location, date and sampling procedures.

Novel plant genotypes

Describe the methods by which all novel plant genotypes were produced. This includes those generated by transgenic approaches, gene editing, chemical/radiation-based mutagenesis and hybridization. For transgenic lines, describe the transformation method, the number of independent lines analyzed and the generation upon which experiments were performed. For gene-edited lines, describe the editor used, the endogenous sequence targeted for editing, the targeting guide RNA sequence (if applicable) and how the editor was applied.

Authentication

Describe any authentication procedures for each seed stock used or novel genotype generated. Describe any experiments used to assess the effect of a mutation and, where applicable, how potential secondary effects (e.g. second site T-DNA insertions, mosiacism, off-target gene editing) were examined.
